# Radiographic and Clinical Outcomes following Combined Oblique Lumbar Interbody Fusion and Lateral Instrumentation for the Treatment of Degenerative Spine Deformity: A Preliminary Retrospective Study

**DOI:** 10.1155/2019/5672162

**Published:** 2019-01-08

**Authors:** Kai Wang, Can Zhang, Cheng Cheng, Fengzeng Jian, Hao Wu

**Affiliations:** Department of Neurosurgery, Xuanwu Hospital of Capital Medical University, 100053, China

## Abstract

**Objective:**

The authors recently used a combination of minimally invasive oblique lumbar interbody fusion (OLIF) and lateral fixation for the treatment of degenerative spine deformity. The early results were promising. Radiographic and clinical results as well as complications were retrospectively assessed in the current study.

**Methods:**

Eleven patients with degenerative spine deformity underwent combined OLIF and lateral instrumentation without real-time electromyography (EMG) monitoring. Radiographic measurements including coronal Cobb angle, central sacral vertebral line (CSVL), lumbar lordosis (LL), sagittal vertebral axis (SVA), pelvic tilt (PT), and LL-PI (pelvic incidence) mismatch were taken preoperatively and at last follow-up postoperatively in all patients. Concurrently, the visual analog score (VAS) for back pain and the Oswestry Disability Index (ODI) score were used to assess clinical outcomes. The fusion rate of OLIF cage, total blood loss, operation time, hospital stay, and complications were also evaluated.

**Results:**

At last follow-up, all patients who underwent combined OLIF and lateral instrumentation achieved statistically significant improvement in coronal Cobb angle (from 15.3±4.7° to 5.9±3.1°, p < 0.01), LL (from 34.3±9.0° to 48.2±8.5°, p < 0.01), PT (from 24.2±9.6° to 16.2±6.0°, p < 0.01), LL-PI mismatch (from 15.4±8.7° to 7.0±3.7°, p < 0.01), CSVL (from 2.1±2.2cm to 0.7±0.9cm, p = 0.01), and SVA (from 7.0±3.9cm to 2.9±1.8cm, p < 0.01). VAS for back pain (from 6.9±1.4 to 2.0±0.9, p < 0.05) and ODI (from 39.5±3.1 to 21.9±3.6, p < 0.01) improved significantly after surgery.

**Conclusions:**

A combination of OLIF and lateral instrumentation is an effective and safety means of achieving correction of both coronal and sagittal deformity, resulting in improvement of quality of life in patients with degenerative spine deformity. It is a promising way to treat patients with moderate degenerative spine deformity.

## 1. Introduction

Degenerative spine deformity is an acquired and progressive disease process that increases in frequency and severity with age [1]. The pathogenesis of degenerative spine deformity is multifactorial and the mechanism is related to progressive degenerative disc disease, compression fractures, disorders of bone quality, and osteoarthritis that creates an asymmetrical deformity of the spine in the coronal and sagittal planes [2–4]. The most common symptom is chronic back pain, neurogenic claudication, radiculopathy, and in later stages significant physical limitations and emotional distress [5–7].

Surgical treatment of degenerative spine deformity is extremely variable and ranges from limited minimally invasive procedures to extensive and lengthy multistaged operations [3, 4, 8, 9]. Traditional open approaches have been the mainstay treatment for degenerative spine deformity. However, the minimally invasive transpsoas lateral lumbar interbody fusion (LLIF) approach is playing an increasingly important role in the treatment of degenerative spine deformity in past decade [10, 11]. It has been reported to be effective in neural decompression and providing some correction of degenerative lumbar scoliosis with less blood loss and morbidity than traditional open procedures [3, 12–18].

However, LLIF is associated with direct muscle injury and a risk of injury to the lumbar plexus as it courses through the psoas [19, 20]. Furthermore, high rates of transient anterior thigh symptoms are found despite real-time electromyography (EMG) monitoring [21]. The oblique lateral interbody fusion (OLIF) was introduced as an alternative procedure to the transpsoas approach, allowing for psoas preservation, and avoids the lumbar plexus [22, 23]. Moreover, OLIF with posterior instrumentation has shown its effectiveness in degenerative spine deformity correction [24]. However, this procedure needs two different approaches, adding more risks and economic expense. On the other hand, it has been reported that OLIF using lateral instrumentation is a feasible, effective, and safe means in treating lumbar degenerative diseases via same approach in one stage surgery [25]. To the best of our knowledge, there has been no report on results and complications of such construct in treating degenerative spine deformity. The purpose of this study is to analyze the clinical and radiographic efficacy of combined OLIF and lateral instrumentation in treating degenerative spine deformity and to determine any complications that occur during operation. The preliminary outcome was promising.

## 2. Materials and Methods

### 2.1. Study Population

This is a retrospective study. A total of 11 patients in Xuanwu Hospital between May 2017 and February 2018 were included in this study based on the following inclusion criteria: (1) diagnosis of degenerative scoliosis (coronal Cobb angle > 10° or sagittal vertebral axis > 5 cm); (2) treatment of adult degenerative scoliosis with OLIF and lateral instrumentation through the same approach in one stage; (3) availability of preoperative and postoperative 36-inch films of the scoliosis. Patient demographics are summarized in Table 1.

### 2.2. Surgical Techniques

The general technique of OLIF has been previously described [22, 23]. An appropriately sized polyetheretherketone (PEEK) interbody cage (DePuy Synthes, Raynham, MA, USA) filled with allogeneic bone graft and hydroxyapatite was inserted into the disc space, creating proper lumbar lordosis. In this study, the spine was approached from the concavity of the scoliotic curve with the intention to approach several levels through a single incision. In cases without significant coronal plane deformity, a left-sided approach was preferred because of the resilience and more ventral location of the aorta over that of the great veins. After OLIF procedure, monoaxial screws with vertebral staples with the shape of a washer with spikes to improve the stability of the screw on the vertebral body were placed at the lateral part of vertebrae. The screws were usually inserted upward and downward so that segmental vessels would be spared. The rods were then placed with compression, cantilever, and rotation to achieve coronal plane correction as well as sagittal plane. Contouring of the rods is almost always required in order to match the physiologic lordotic spinal curve. No patient received a supplementary posterior instrumentation in a second stage. All patients were allowed to ambulate by Boston brace on the second postoperative day. The Boston brace was recommended for removal after 12 weeks.

### 2.3. Radiographic and Clinical Evaluation

**Figure 1:**
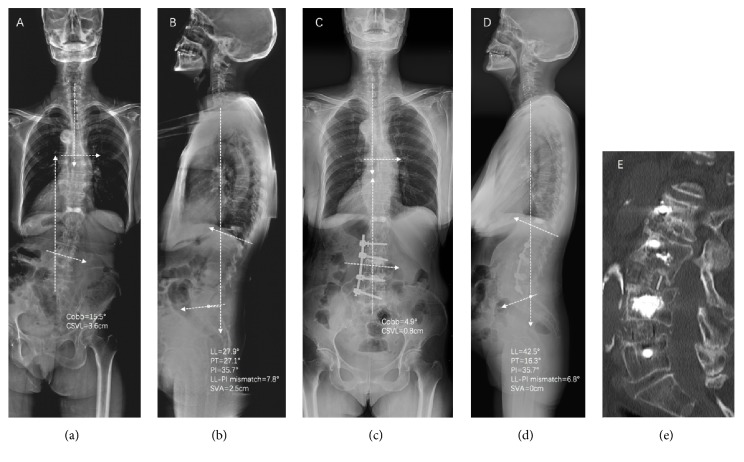
Preoperative standing AP (a) and lateral (b) 36-inch films and postoperative standing AP (c) and lateral (d) 36-inch films of patient 3 who underwent a combined OLIF and lateral procedure for correction of degenerative spine deformity. A rod was contoured in order to match the physiologic lordosis of lumbar spine (d). A satisfactory correction in coronal Cobb angle, LL, PT, LL-PI mismatch, CSVL, and SVA was achieved. Parameters of coronal plane (a, c) and sagittal plane (b,d) were illustrated.

**Figure 2:**
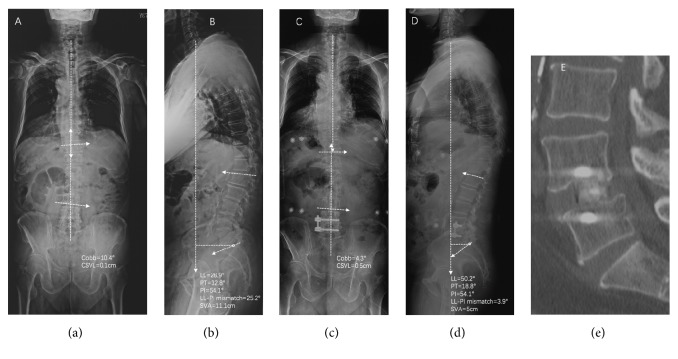
Preoperative standing AP (a) and lateral (b) 36-inch films and postoperative standing AP (c) and lateral (d) 36-inch films of patient 5 who underwent a combined OLIF and lateral procedure for correction of degenerative spine deformity. OLIF procedure was performed at L4-5 where degenerative change of intervertebral space happened. However, the coronal and sagittal spine alignment has been improved.

**Figure 3:**
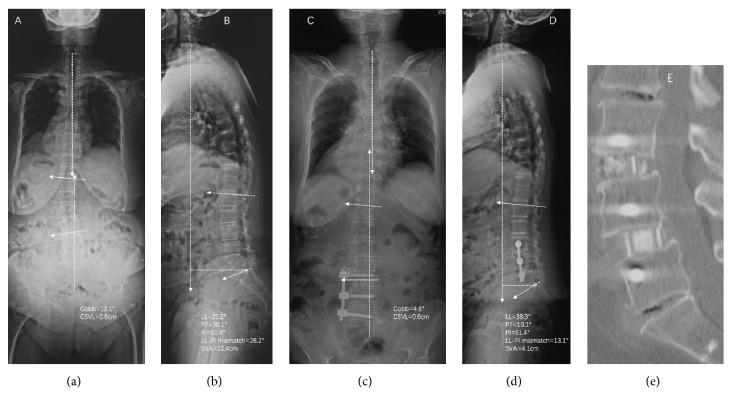
Preoperative standing AP (a) and lateral (b) 36-inch films and postoperative standing AP (c) and lateral (d) 36-inch films of patient 9 who underwent a combined OLIF and lateral procedure for correction of degenerative spine deformity.

Standing anteroposterior and lateral 36-in films were obtained in 11 patients preoperatively and at last follow-up for measurement (Figures 1–3). The coronal Cobb angle, central sacral vertebral line (CSVL), lumbar lordosis (LL), sagittal vertebral axis (SVA), pelvic tilt (PT), and LL-PI (pelvic incidence) mismatch were determined and compared. Computed tomography images obtained at last follow-up were reviewed to assess bridging bone to determine if bony fusion had occurred. Fusion criteria on CT studies included the presence of bony trabeculation across the fusion level and lack of bony lucency at the graft/vertebral body junction [26–28].

Lower back pain was evaluated according to the visual analog scale (VAS). The Oswestry Disability Index (ODI) before surgery and at last routine postoperative clinic visits were compared. Surgical characteristics and complications were also recorded (Table 1).

Complications were evaluated at last follow-up and retrospectively reviewed in this study.

### 2.4. Statistical Analysis

Statistical analysis was calculated using the paired t-test; a p value ≤ 0.05 was considered statistically significant.

## 3. Results

A total of 11 patients (1 man and 10 women) were included in the study (Table 2). The mean patient age was 71.5±10.1 years (range 56–86 years). All patients had a diagnosis of degenerative spine deformity and were successfully treated with combined OLIF and lateral instrumentation. Demographic and operative characteristics of the patients were shown in Table 1.

Coronal Cobb angle, CSVL, LL, PT, LL-PI mismatch, and SVA were significantly improved at the final follow-up compared with the values before surgery (p<0.05, Table 3). Fusion was seen at all of the OLIF levels (Table 1).

Lower back pain evaluated by VAS was significantly improved from 6.9±1.4 before surgery to 2.0±0.9 at last follow-up after surgery (P<0.05). ODI was also significantly improved from 39.5±3.1 to 21.9±3.6 (P<0.05).

The OLIF procedures in the lumbar spine are associated with transient or permanent symptoms in the thigh. Due to retrospective direction of observation, these data were not consistently available and thus were not included in the study. However, thigh numbness, pain, dysesthesias, or weakness indicative of a lumbosacral plexopathy was not seen in any of the patients by their last follow-up visit. Cage subsidence at 2 levels was observed in 2 of the patients by their last follow-up. There were no other major or minor intraoperative or perioperative complications. Hardware failure did not occur.

## 4. Discussion

Minimally invasive spinal fusion in particular has been associated with decreased blood loss, decreased postoperative pain, and shorter hospital stays compared with traditional open procedures [17, 29]. Minimally invasive fusion techniques are being continually refined and allow the surgeon to gain access to both the anterior and posterior spinal columns with smaller incisions and less tissue disruption. The LLIF can access to the anterior lumbar disc space and allows for discectomy, release, and interbody fusion [30–32]. The utility of this technique in the treatment of spinal deformity, specifically with respect to adult degenerative scoliosis, has also been evaluated and reported excellent results for deformity correction in both coronal and sagittal planes [3, 13, 16–18, 33, 34].

The interbody cage used in LLIF surgery distracts the anterior and middle columns of the lumbar spine, restoring lost disc space height and achieving indirectly decompression. Besides, LLIF surgery has the capability of improving lordosis [17, 18, 35–37]. Additionally, the LLIF operation allows direct manipulation of the anterior and middle columns, which permits a potentially greater degree of deformity correction compared with manipulation from a posterior approach alone [3]. Neural decompression and restoration of sagittal and global spinal balance lead to decrease in pain and increase in quality of life measures [38, 39]. Moreover, LLIF results in less blood loss, overall operative time, length of hospital stay, and complication incidence than traditional open procedures in patients with degenerative spine deformity [14, 15].

However, some author reported complications LLIF. Symptoms included pain, numbness, paresthesias, or weakness due to direct muscle injury and injury to the lumbar plexus as it courses through the psoas [19, 20]. Furthermore, high rates (62.7%) of transient anterior thigh symptoms are found despite real-time electromyography (EMG) monitoring [21]. Ohtori et al. have reported OLIF surgery for degenerated lumbar spinal kyphoscoliosis without real-time EMG monitoring [4]. The surgical results were good and few motor or sensory nerve injury or symptoms from the psoas muscle were observed. Thus, they concluded that OLIF was capable of correcting degenerative spine deformity while avoiding the reported complications of LLIF procedure. However, the procedure reported needs posterior fixation through a different approach in the same or a second stage surgery, adding more operation time, blood loss, risks, and economic expense. Lin et al. combined OLIF and lateral instrumentation to treat anterior lumbar disease and reported satisfactory clinical outcome [25].

In our study, combined OLIF and lateral instrumentation has shown its advantage in treating degenerative spine deformity. OLIF procedure could restore the lost disc space height and achieve indirectly neural decompression as LLIF [24]. Furthermore, as the mechanism of degenerative spine deformity is related to progressive degenerative disc disease and osteoarthritis that may create an asymmetrical loss of the height of intervertebral space, OLIF could restore the sagittal and global spinal alignment by leveling the disc space and the vertebrae intended to fuse [3, 4]. Then the lateral fixation with pre-bent rod could achieve further coronal and sagittal plane correction. The mean coronal Cobb angle, LL, PT, and LL-PI mismatch were improved and the coronal and sagittal alignment in this study was restored as reported in other studies [3, 4, 13, 16–18, 34, 36, 40]. The pain scores and quality of life measures were also improved as reported [3, 4, 13, 17]. Meanwhile, average operation time and average blood loss during surgery were observed to be 144.0±50.0 minutes and 94.5±72.4mL, which were less compared to OLIF with posterior fixation [4].

However, the current study has some limitations. First of all, it is a small sized study and the duration of follow-up was also short. Moreover, no severe sagittal imbalance case, such as lumbar kyphosis or flat back, was included in our study. Dakwar found that in one-third of patients sagittal plane correction was not adequate after they had undergone lateral interbody fusion for degenerative spine deformity [16]. Traditionally, the primary methods for correcting sagittal imbalance have been the pedicle subtraction osteotomy (PSO) and Smith-Petersen osteotomy (SPO) [41–43]. Recently, Deukmedjian et al. reported several cases of minimal invasive (MI) anterior column release (ACR) as a means of correcting sagittal imbalance via a LLIF approach [44]. The MI placement of posterior instrumentation is necessary when an ACR is performed and can be used to obtain further correction [17, 18]. Thus, for severe sagittal imbalance cases, OLIF combining lateral instrumentation may not be adequate in sagittal plane correction.

## 5. Conclusions

OLIF combining lateral instrumentation seems to be a valuable surgical tool for the minimally invasive correction of coronal and sagittal plane deformities in patients with moderate degenerative spine deformity. It is a less invasive procedure to achieve good radiographic and clinic results without any major complications.

## Figures and Tables

**Table 1:** Summary of patient demographics and surgical characteristics.

Parameter	Value
Total no. of patients	11
Sex ratio (M: F)	1:11
Age, mean (range), years	71.5±10.1 (56-86)
BMI, mean (range), Kg/m^2^	27.2±2.9 (21.9-31.6)
OLIF Level	
L4-5	2
L2-4	3
L3-5	2
L2-5	4
Mean no. of previous caudal fusion levels	1.5 ±0.5
Operation time, mean (range), mins	144.0±50.0 (74-237)
Blood loss, mean (range), ml	94.5±72.4 (20-200)
Hospital stay, mean (range), days	4.1±1.6 (2-6)
Follow-up after surgery, mean (range), months	9.7±2.8 (6-15)
Fusion rate (CT confirmation)	100%

**Table 2:** Data of all patients.

Patient	Sex	Age (years)	Height (m)	Weight (Kg)	BMI (Kg/m^2^)	Cobb Pre (°)	Cobb Post (°)	CSVL Pre (cm)	CSVL Post (cm)	PI(°)	PT Pre(°)	PT Post(°)	LL Pre(°)	LL Post(°)	SVA Pre (cm)	SVA Post (cm)	LL-PI Mismatch Pre (°)	LL-PI Mismatch Post (°)	VAS Pre	VAS Post	ODI Pre	ODI Post
1	F	75	1.54	75	31.6	13.3	9.1	1.7	0	52.5	16.0	14.3	41.2	59.6	4	3.9	11.3	7.1	7	1	33	22
2	F	82	1.59	70	27.7	13.5	4.6	1.2	0	55	29.0	23.0	46.0	56.0	8.5	4.7	9	1	10	3	43	26
3	F	78	1.60	56	21.9	15.5	4.9	3.6	0.8	35.7	27.1	16.3	27.9	42.5	2.5	0	7.8	6.8	5	1	40	15
4	F	63	1.55	75	31.2	10.3	3.9	0.7	0.7	45.4	19.1	14.7	28.7	36.5	6.92	1.82	16.7	8.9	7	2	37	22
5	M	73	1.65	75	27.5	10.4	4.3	1.1	0.7	54.1	32.8	18.8	28.9	50.2	11.1	5	25.2	3.9	6	1	39	27
6	F	57	1.55	62	25.8	18.3	9.2	2.3	1.1	62.5	33.0	20.4	43.1	53.9	10.1	4.7	19.4	9.6	6	1	41	18
7	F	56	1.64	67	24.9	19	8.1	1.1	0	43.5	5.3	9.1	40.8	46.5	0	1.21	2.7	3	6	2	42	21
8	F	68	1.60	70	27.3	20.5	3.8	8.4	3.1	50.9	36.2	25.5	21.0	39.2	6.5	0.6	29.9	11.7	6	2	40	21
9	F	86	1.50	60	26.7	13.1	4.6	0.6	0	51.4	30.1	19.1	25.2	38.3	12.4	4.1	26.2	13.1	8	3	40	26
10	F	68	1.50	65	28.9	10.1	1	1.7	1.1	55.5	23.9	11.9	45.5	60.2	4.7	2.6	10	4.7	7	3	36	20
11	F	81	1.60	63	24.6	24.5	11.2	1	0.5	40.1	14.0	5.5	29.0	46.8	9.9	3.5	11.1	6.7	8	3	43	23

**Table 3:** Radiographic outcomes.

Variable	Preoperative	Postoperative	*P*
Coronal Cobb Angle (°)	15.3±4.7	5.9±3.1	0.00
LL (°)	34.3±9.0	48.2±8.5	0.00
PT (°)	24.2±9.6	16.2±6.0	0.00
LL-PI Mismatch (°)	15.4±8.7	7.0±3.7	0.00
CSVL (cm)	2.1±2.2	0.7±0.9	0.01
SVA (cm)	7.0±3.9	2.9±1.8	0.00

## Data Availability

The radiographic and clinical data used to support the findings of this study are included within the article.
